# Freezing: how do water mites (Acari: Hydrachnidia) survive exposure to sub-zero temperatures?

**DOI:** 10.1007/s10493-021-00634-2

**Published:** 2021-06-21

**Authors:** Andrzej Zawal, Tomasz Czernicki, Grzegorz Michoński, Aleksandra Bańkowska, Robert Stryjecki, Vladimir Pešić, Magdalena Achrem, Jakub Skorupski, Joanna Pakulnicka, Agnieszka Szlauer-Łukaszewska

**Affiliations:** 1 Institute of Marine and Environmental Sciences, Center of Molecular Biology and Biotechnology, University of Szczecin, Szczecin, Poland; 2 Institute of Biology, Center of Molecular Biology and Biotechnology, University of Szczecin, Szczecin, Poland; 3 Department of Zoology, Animal Ecology and Wildlife Management, University of Life Sciences in Lublin, Lublin, Poland; 4 Department of Biology, University of Montenegro, Podgorica, Montenegro; 5 Department of Ecology and Environmental Protection, University of Warmia and Mazury in Olsztyn, Olsztyn, Poland

**Keywords:** Water mite species, Survival strategies, Overwintering, Influence of acclimatization, Lethal temperature

## Abstract

Until now, very little is known about the ability of adult and deutonymph water mites (Acari, Hydrachnidia) to survive in sub-zero temperatures. Information concerns mainly water mites from vernal astatic waters, and the knowledge has never been experimentally verified. To determine the sensitivity of water mites to freezing, experiments were conducted on (1) the impact of acclimatization, (2) temperature, and (3) duration of freezing on survival, (4) the survival rate of water mites from various types of water bodies, and (5) the survival rate of water mites from different climatic zones. The experiments were carried out in a phytotron chamber, and water mites were placed in containers (10 × 10 × 5 cm) filled with 4/5 of water for 10 specimens each. Water mites were identified to the species level after finishing the experiments. The temperature was lowered 1 °C every hour until the target temperature was reached. After a certain period of freezing (depending on the treatment) the temperature was raised by 1 °C every hour until it reached 4 °C. The time of the experiment was measured from the moment the desired temperature was reached (below 0 °C) until the ice thawed and the temperature of 4 °C was reached again. The highest survival rates had *Limnochares aquatica*, *Piona nodata*, *Sperchon clupeifer* and *Lebertia porosa*, followed by *L. insignis*, *Hygrobates longipalpis*, *H. setosus*, *Limnesia undulatoides*, *Piona pusilla*, *Arrenurus globator*, *Hydrodroma despiciens*, *Piona longipalpis*, *Sperchonopsis verrucosa*, *Unionicola crassipes* and *Mideopsis crassipes*; no specimens of *Torrenticola amplexa* survived. The following conclusions were drawn: (1) water mites can survive freezing to −2 °C, lower temperatures are lethal for them; (2) they survived better the short period of freezing (24–48 h) than the long period (168 h); (3) resistance to freezing seems to be an evolutionary trait of individual species, only partly related to the living environment; and (4) freezing survival rates are linked to the region of Europe and are much lower in Southern than in Central Europe.

## Introduction

Exposure to low temperatures is among the most important abiotic factors limiting the range of invertebrates in temperate climates (Rivers 2008). Low temperatures force animals to develop adaptations enabling them to survive in specific temperature conditions. Cold-blooded organisms must be adapted to survive low temperatures in winter in order to maintain sustainable populations in regions with temperate and cold climates. Invertebrates exposed to sub-zero temperatures can be divided into two groups: freezing-tolerant and freezing-intolerant strategies (Frisbie and Lee 1997; Rivers 2008).

Analysis of the problem of adaptation of invertebrates to sub-zero temperatures should distinguish terrestrial and aquatic ecosystems. Terrestrial and aquatic environments differ in terms of the possibility, frequency and scale of effects of sub-zero temperatures on animals. Thus, there is a difference in resistance to low temperatures between terrestrial and aquatic invertebrates. Generally, terrestrial invertebrates can tolerate much lower temperatures than aquatic ones, and semi-terrestrial (mosses and soil) tardigrades can survive extremely low temperatures (Somme 1996). Many species of terrestrial insects tolerate high sub-zero temperatures (Rivers 2008).

In the aquatic environment, the specific physical properties of the water cause different reactions to low temperatures in invertebrates and lead to a variety of adaptations. Due to water has a high heat capacity, organisms living in it are much less susceptible to extremely low temperatures than terrestrial once. On the other hand, freezing of water may cause damage of internal tissues, and the presence of external ice may cause internal ice in their bodies. This is the main difference between terrestrial organisms and aquatic ones, which are able to avoid internal freezing by supercooling, i.e., remain unfrozen at temperatures below the freezing point of their body fluids (Frisbie and Lee 1997).

Many aquatic invertebrates have been studied with respect to low-temperature tolerance (Frisbie and Lee 1997), but water mites (Acari, Hydrachnidia) have been scarcely studied. Water mites are common and widespread aquatic invertebrates present in nearly every type of aquatic ecosystem (Di Sabatino et al. 2000, 2003; Davids et al. 2007). Many species occur in temperate and cold climates (Davids et al. 2007; Di Sabatino et al. 2010; Gerecke et al. 2016). There are some works dealing with overwintering of water mites (Gerecke 2002; Martin 2010). Some (Gerecke et al. 2005; Gerecke and Martin 2006; Zawal and Szlauer-Łukaszewska 2012) mention that certain water mite species are able to colonize extreme habitats which are potentially subject to freezing, especially vernal temporary water bodies, where water mites survived freezing in substratum at the bottom of dry reservoirs (Wiggins et al. 1980; Smith 1987). But there is no detailed information about the ability of water mites to survive freezing temperatures, and no experimental work has been carried out on this topic.

To avoid exposure to sub-zero temperatures and potential freezing in shallow water during the winter, in larger water bodies (lakes) water mites migrate into deeper parts where the temperature never drops below 0 °C (Pieczyński 1964; Kowalik 1973; Zawal et al. 2014). This is an example of behavioural adaptation and a freezing-intolerant strategy, which indicates that lake water mites could be classified as freeze-avoiding animals. However, this strategy cannot be applied in every habitat. In some habitats, such as small water bodies, peatland water bodies, subterranean waters, or extreme habitats at high altitudes, water mites may be exposed to freezing temperatures. Are these water mites freezing-tolerant?

The aim of this work was to determine whether water mites of various species are able to survive freezing (sub-zero) temperatures, and whether their survival depends on the temperature value (degrees below zero) and duration of freezing.

## Material and methods

In total, 2452 water mite specimens belonging to 50 species were used in the experiments. They were collected from April to November from the following water bodies in Poland and Montenergro. In Poland, (1) Siecino Lake (53°37′58.4″N, 16°00′51.7″E), (2) Czermnica Lake (53°42′46.4″N, 14°57′41.0″E), (3) Węgorza Lake (53°40′22.8″N, 14°58′14.6″E), (4) Jaworowy Pond in Szczecin (53°28′35.0″N, 14°32′43.2″E), (5) Łomot Pond in Szczecin (53°28′56.1″N, 14°30′27.3″E), (6) the pond in the village of Moczyły (53°19′28.8″N, 14°28′14.0″E), (7) the river Tywa near the village of Żurawki (53°13′36.7″N, 14°29′19.9″E), (8) the river Krąpiel near the village of Ulikowo (53°19′13.0″N, 15^o^6′4.52″E), and (9) the spring (helocrene) near the village of Ulikowo (53°19′13.0″N, 15^o^6′4.52″E). In Montenegro, (10) Skadar Lake (42°14′44.5″N, 19°05′42.8″E), (11) the river Zeta (42°30′42.4″N, 19°11′48.2″E), and (12) Crno Oko Spring (limnocrene) (42°29′04.2″N, 19°09′16.1″E).

Water mites from the depth down to 1.5 m were collected using a hydro-biological sampler (hand-net) with a triangular hoop. Water mites from greater depths were collected using a dredge with a triangular hoop. Water mites were sorted immediately after collecting and were delivered into laboratory in water from the sampling point. Water mites were divided into groups suitable for experiments, placed in 1-L containers in pure tap water in temperature similar to the temperature of the sampled water, and stored at this temperature until the next day, when the experiment was started. Each specimen was used only once and only in one experiment. As the identification of live water mites to the species level is possible only to a certain extent, mites were divided into morphologically similar groups, which were subjected to experiments in separate containers, and the final species identification was made after the experiment was completed and microscopy slides were mounted, using the keys of Tuzovskij (1990), Davids et al. (2007), Di Sabatino et al. (2010) and Gerecke et al. (2016).

Water mites from Montenegro were transported to Poland by car right after collection, in a portable refrigerator in the temperature range 7–10 °C, The duration of the transport was 26 h. Upon arrival, they were immediately acclimated and the experiment began.

For the purpose of the experiments, 10 specimens of water mites were placed in containers with dimensions 10 × 10 × 5 cm filled for 80% with water. Because water mites are known to withstand periods of hunger up to 30 days without obvious symptoms (Bańkowska et al. 2016), and their feeding could introduce additional elements of uncertainty, it was decided not to feed them for the entire preparatory and experiment period.

The experiments were carried out in a phytotron chamber. The temperature was lowered 1 °C every hour from 4 °C (exception non-acclimated water mites in experiment 1) until the target temperature was reached. After a certain period of freezing (depending on the treatment) the temperature was raised by 1 °C every hour until it reached 4 °C. The time of the experiment was measured from the moment the desired temperature was reached (below 0 °C) until the ice thawed and the temperature of 4 °C was reached again.

To determine the sensitivity of water mites to freezing, five experiments were conducted, investigating: (1) the impact of acclimatization on survival, (2) the impact of temperature on survival, (3) the impact of freezing duration on survival, (4) the survival rate of particular species collected in various months (April–November) and various types of water body (permanent small ponds, lakes, rivers and springs), and (5) the survival rate of water mites from various climatic zones.

The results of each experiment determined the conditions of the subsequent ones. The results of experiment 1 determined the use of acclimated water mites in all subsequent experiments. The results of experiment 2 determined the subsequent use of −1 and −2 °C. The results of experiment 3 determined the freezing time of 24 h. In experiments 1 and 2, water mites were tested in three variants: low temperature (below 0 °C) in water and in wet conditions (on tissue paper), and high temperature (4 °C) in water (control). In the other experiments water mites were tested in two variants: low temperature (below 0 °C) in water and high temperature (4 °C) in water (control). All experiments were performed in triplicate, with three groups of water mites for each variant. Each test group comprised maximum of 10 specimens in one container. Thus, the experiments were conducted under the following conditions:

Experiment 1: Acclimated and non-acclimated groups of water mites were placed in water at two temperatures (−1 and 4 °C) for 24 h. Acclimatization was done by placing the mites in water of 4 °C for 1 week. In this time non-acclimated mites were kept in temperature similar to the temperature of water from the sampling place, e.g., 15 °C.

Experiment 2: Water mites were tested at low temperatures of −1, −2, −3, −5 and −8 °C for 24 h. In total, 672 specimens belonging to 18 species (see Results) were used, but only eight of them exceeded the number of 10 specimens.

Experiment 3: Water mites were tested at −1 °C for 24, 48 and 168 h. In total, 309 specimens belonging to 15 species were used, but only eight species had more than 10 specimens.

Experiment 4: Various species of water mites, collected from different types of water body (lakes, permanent ponds, rivers and springs) and in different months (April–November) were tested at two temperatures (−1 and 4 °C) for 24 h, to check for differences in survival rates. The experiment was performed 8 × , in each month after catching water mites. In total, 1016 specimens belonging to 24 species were used, but only eight had more than 10 specimens.

Experiment 5: Water mites from Montenegro and Poland were tested to check for differences in survival among climatic zones. The water mites were kept at −1 °C for 24 h. In total, 225 specimens belonging to five species collected in Montenegro were compared with the same species (110 specimens) from Poland, tested in experiments 2 and 4 in water of −1 °C. Among these species, *Hygrobates fluviatilis* and *Leberetia longiseta* had fewer than 10 specimens.

The results were compared between temperatures and freezing times, survival ratios (number of surviving specimens/total number) were used in all statistical analyses. For single species, survival ratios were calculated only within species; for the whole of water mites, all mites were lumped irrespective of species. The significance of statistical differences (α = 0.05) was assessed by Kruskal–Wallis test and Mann–Whitney U test (STATISTICA v.13 PL).

## Results

In total, 2452 specimens belonging to 50 species of water mites were tested. Most of them were represented mainly by adults, one species (*Piona nodata*) mainly by deutonymphs, and six (*Arrenurus globator*, *Hygrobates longipalpis, Lebertia porosa*, *Limnocharea aquatica*, *Sperchonopsis verrucosa*, *Unionicola crassipes*) by both stages. In almost all cases, adults survived freezing much better than deutonymphs, the only exception was *P. nodata* where the opposite was true (Table 1). Unfortunately, there were many species with fewer than 10 specimens, which could only be seen after the detailed species identification, after finishing the experiments. We include the data on the species present in low numbers, although we realize that the information is uncertain.

**Table 1:** Water mite species used for researches of surviving the state of freezing (a/d, adults/deutonymphs)

Species	Number of specimens a/d	Temperature/ratio of survive a/d		Number of locality	Number of experiment
		−2	−1		
*Arrenurus albator* (Müller)	1/0	–	–	1	2
*Arrenurus bifidicodulus* Piersig	8/1	0.17/0.00	–	2, 5	2, 3, 4
*Arrenurus bruzelii* Koenike	1/0	0.00	–	2	4
*Arrenurus buccinator* (Müller)	1/0	1.00	–	1	4
*Arrenurus claviger* Koenike	7/0	–	0.43	3, 4	2, 3
*Arrenurus cuspidator* (Müller)	4/0	0.50	0.00	6	4
*Arrenurus falciger* K. Viets	8/0	–	0.00	1	4
*Arrenurus fimbriatus* Koenike	3/0	1.00	–	3	4
*Arrenurus globator* (Müller)	14/8	0.00/-	0.15/0.07	3, 4, 5	1, 2
*Arrenurus integrator* (Müller)	1/0	–	–	1	4
*Arrenurus maculator* (Müller)	5/0	0.00	0.33	4	2, 3
*Arrenurus securiformis* Piersig	1/0	–	–	1	1
*Arrenurus tubulator* (Müller)	4/0	–	0.75	3	1
*Atractides ovalis* Koenike	2/1	–	0.00/0.00	1	4
*Axanopsis complanata*	1/0	–	1.00	1	4
*Forelia liliacea* (Müller)	2/0	–	1.00	2	1
*Huitfeldtia rectipes* Thor	2/0	–	0.00	1	4
*Hydrodroma despiciens* (Müller)	14/1	1.00/0.00	0.17	2, 3	2, 3
*Hydrodroma pilosa* Besseling	1/0	–	–	6	1
*Hygrobates fluviatilis* (Ström)	8/0	0.00	0.50	8, 11	2, 5
*Hygrobates longipalpis* (Hermann)	57/35	1.00/-	0.31/0.05	1, 7, 8, 11, 12	1, 2, 3, 4, 5
*Hygrobates nigromaculatus* Lebert	10/0	–	1.00	8	4
*Hygrobates setosus* Besseling	190/1	0.00	0.41/0.00	7, 8, 9, 11, 12	1, 2, 4, 5
*Lebertia insignis* Neuman	19/3	–	0.44	8, 9	2, 4
*Lebertia longiseta* Bader	4/0	–	0.00	8, 10	4, 5
*Lebertia porosa* Thor	41/17	0.25/-	0.53/0.03	1, 8, 9, 10, 11, 12	1, 2, 3, 4, 5
*Lebertia rivulorum* K. Viets	7/0	1.00	–	7	4
*Limnesia connata* Koenike	4/0	1.00	–	2, 3	1
*Limnesia fulgida* Koch	1/0	1.00	–	1	1
*Limnesia maculata* (Müller)	7/0	–	0.00	5, 6	3
*Limnesia undulata* (Müller)	1/0	–	0.00	4	1
*Limnesia undulatoides* Davids	13/0	–	0.43	4, 5	1, 2, 4
*Limnochares aquatica* (L.)	152/29	1.00/0.00	0.82/0.24	2, 3, 4, 5	1, 2, 3, 4
*Midea orbiculata* (Müller)	3/0	–	1.00	4	2
*Mideopsis crassipes* Soar	25/0	–	0.13	7, 8	2, 3, 4
*Mideopsis orbicularis* (Müller)	6/0	–	0.17	1	1, 2
*Mideopsis roztoczensis* Biesiadka & Kowalik	6/0	–	0.00	8	4
*Neumania callosa* (Koenike)	1/0	–	0.00	8	1
*Piona coccinea* (Koch)	1/0	–	-	5	1
*Piona conglobata* (Koch)	2/0	–	1.00	6	1
*Piona longipalpis* (Krendowskij)	11/0	–	0.29	1, 2, 3	1, 2
*Piona pusilla* (Neuman)	29/3	–	0.25/0.00	4, 5	2, 3
*Piona nodata* (Müller)	25/841	0.00/0.81	0.07/0.58	3, 4, 5, 6	1, 2, 3, 4
*Piona variabilis* (Koch)	6/1	–	0.50/0.00	6	1, 2
*Sperchon clupeifer* Piersig	97/5	0.94/-	0.50/0.00	7, 8, 9	1, 2, 3, 4
*Sperchonopsis verrucosa* (Protz)	24/13	0.00	0.13/0.05	7, 8	1, 2, 3, 4
*Torrenticola amplexa* (Koenike)	50/4	–	0.00/0.00	7, 8	1, 2, 3
*Unionicola crassipes* (Müller)	544/57	0.35/-	0.10/0.00	2, 4, 5, 6, 10	1, 2, 3, 4
*Unionicola gracilipalpis* (K. Viets)	3/0	–	1.00	1	2
*Wettina podagrica* (Koch)	1/0	–	0.00	9	2
Total	1432/1020				

### Experiment 1

**Fig. 1:**
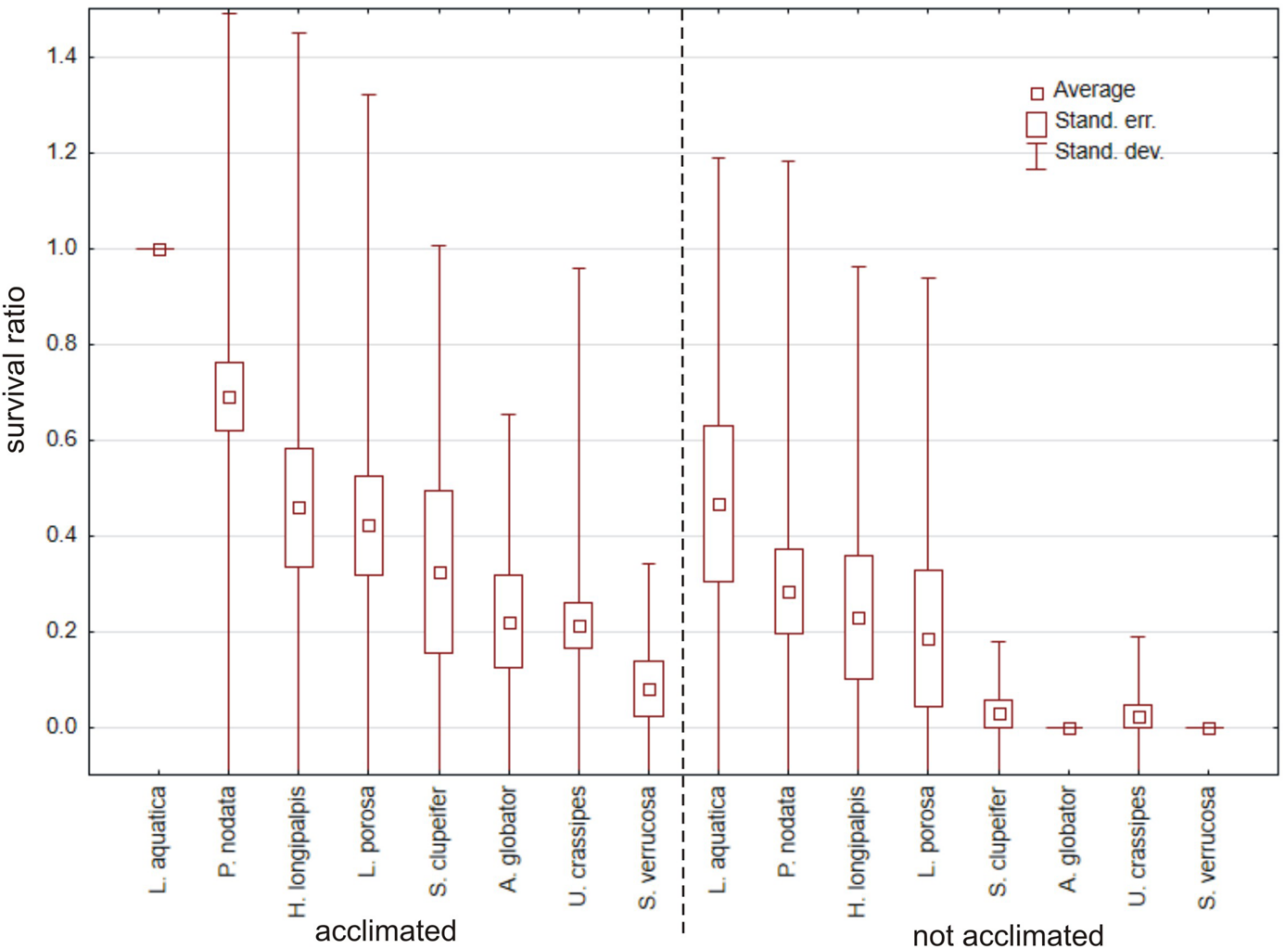
Survival ratio (no. live mites of species X/total no. mites of species X) of freezing (−1 °C) by particular acclimated and non-acclimated species of water mites in water at a temperature −1 °C

**Fig. 2:**
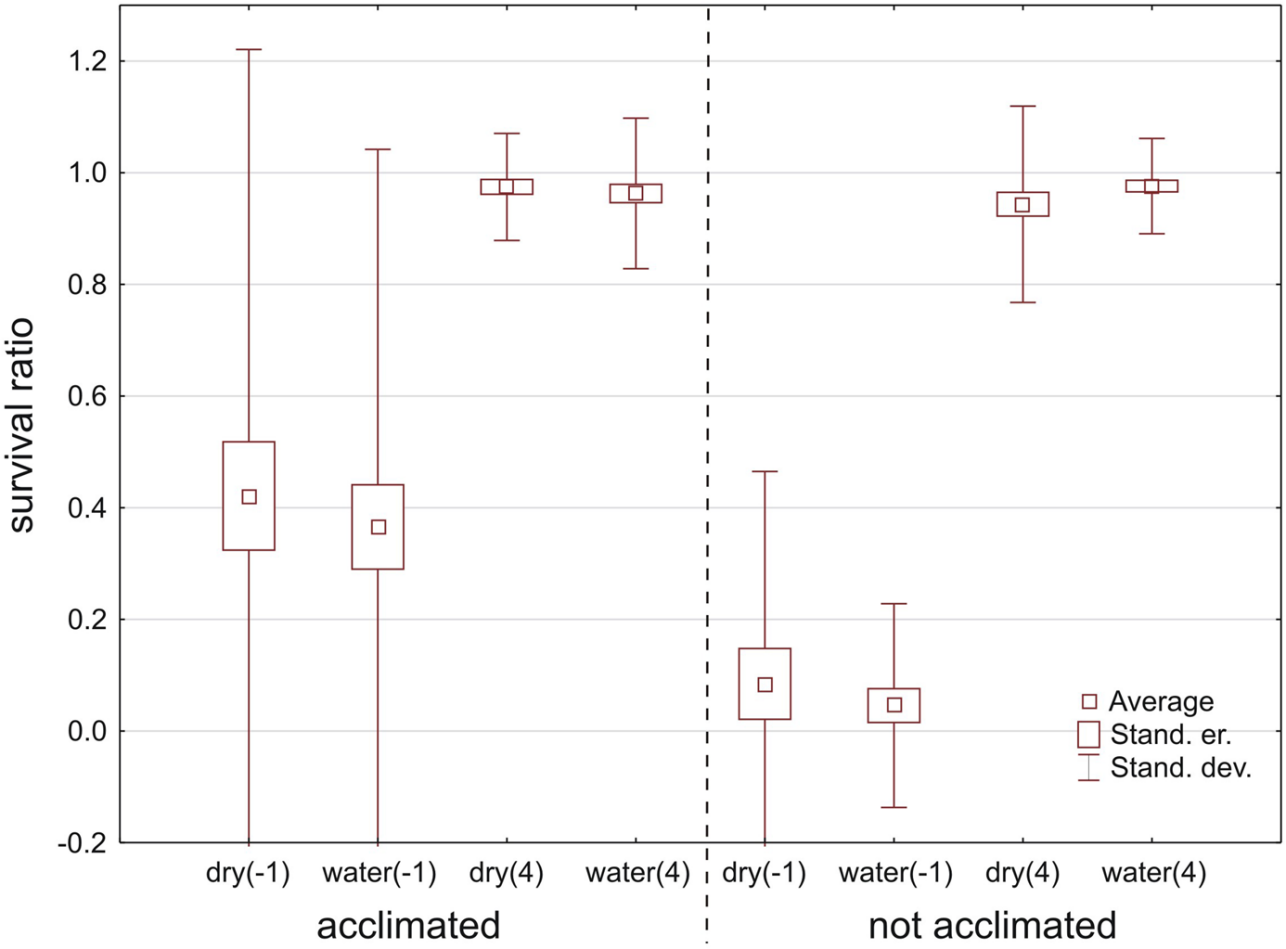
Survival ratio (no. live mites/total no. mites) of freezing (−1 °C) by acclimated and non-acclimated water mites tested on tissue paper [dry(−1)], in water [water(−1)], and in water of 4 °C as a control [water(4)]

**Fig. 3:**
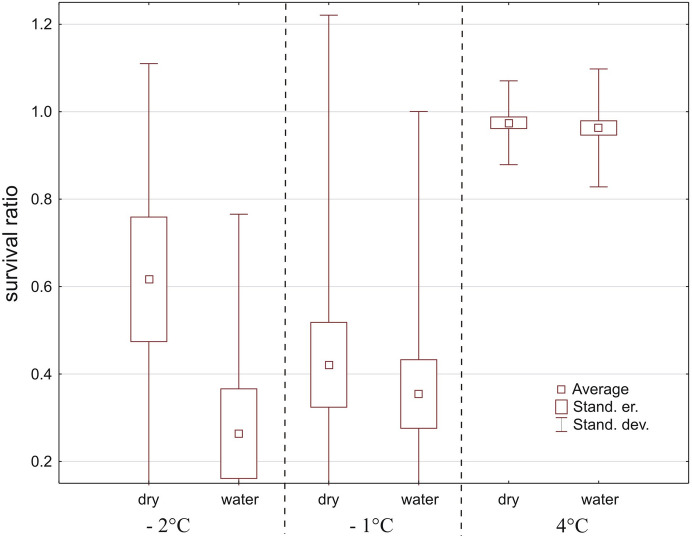
Survival ratio (no. live mites/total no. mites) of water mites at −2, −1 and 4 °C, on tissue paper (dry) or in water

In total, 232 specimens belonging to 26 species (Table 1) were tested, but only eight species had > 10 specimens (Fig. 1). Acclimated water mites survived temperatures below freezing much better than non-acclimated ones, both in water (Mann–Whitney U test: Z = 2.891, *p* = 0.0038) and in wet conditions (Z = 2.781, *p* = 0.0054); survival did not differ between water vs. wet condition (Z < 0.001, *p* = 0.99) (Fig. 2). Among the tested species (Table 1, Fig. 1), the survival ratios differed between acclimated vs. non-acclimated specimens of *Limnochares aquatica* (Z = 2.928, *p* = 0.0034), *Piona nodata* (Z = 2.491, *p* = 0.013), *Sperchon clupeifer* (Z = 1.976, *p* = 0.048), *Arrenurus globator* (Z = 2.138, *p* = 0.033) and *Unionicola crassipes* (Z = 1.982, *p* = 0.048). Survival did not differ between acclimated vs. non-acclimated specimens of *Hygrobates longipalpis* (Z = 1.036, *p* = 0.30), *Lebertia porosa* (Z = 1.180, *p* = 0.24) and *Sperchonopsis verrucosa* (Z = 1.623, *p* = 0.10) (Fig. 3).

### Experiment 2

**Fig. 4:**
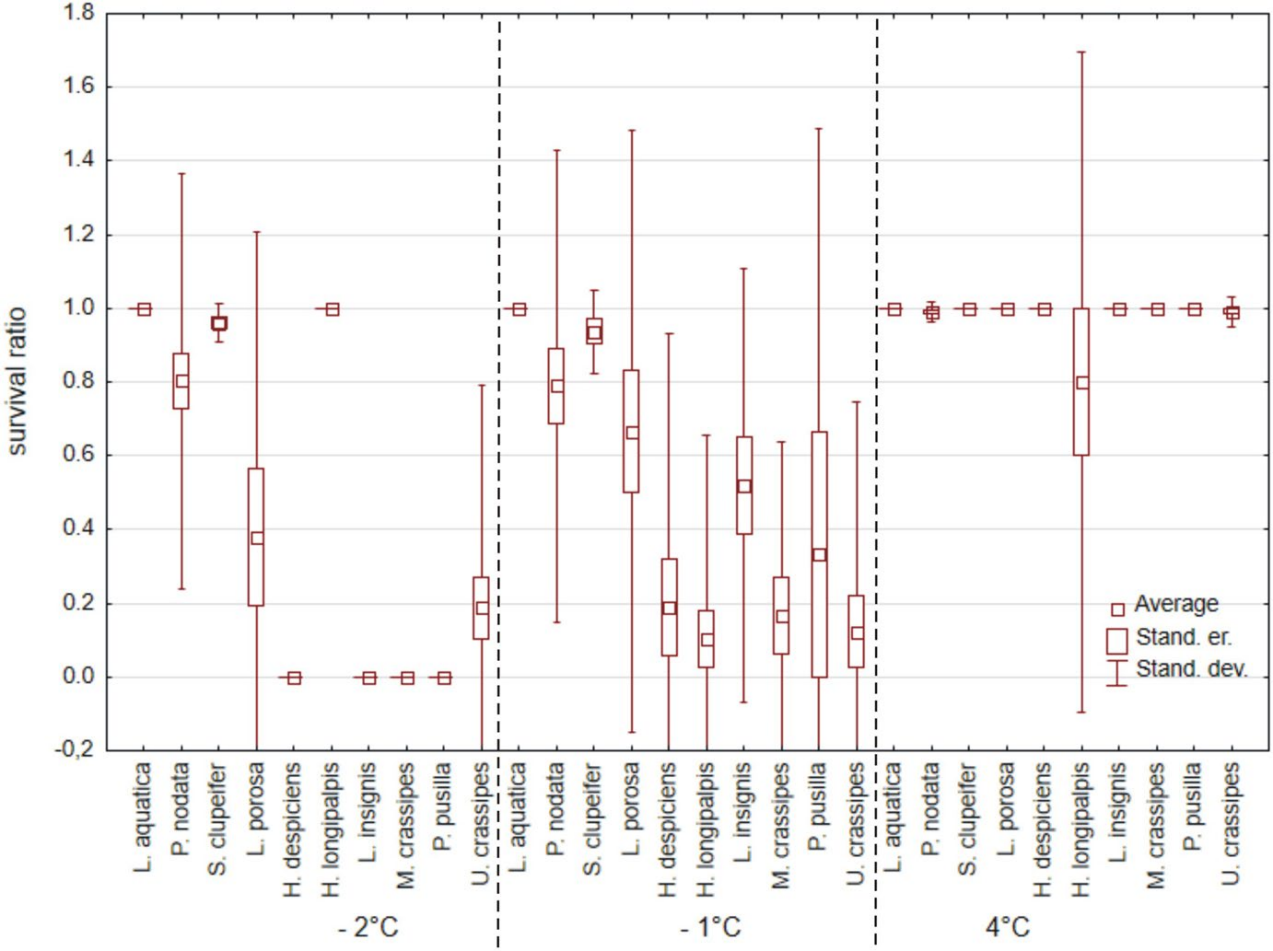
Survival ratio (no. live mites of species X/total no. mites of species X) of the most numerous species of water mites at −2 °C, −1 °C and 4 °C, in water

In total, 672 specimens belonging to 18 species (Table 1) were used, but only eight species had > 10 specimens (Fig. 4). Water mites survived only temperatures of −2 °C and −1 °C, and differences between these temperatures vs. 4 °C (Fig. 4) were significant (Kruskal–Wallis test: H (2, *N* = 40) = 23.838, *p* < 0.0001), whereas differences between −2 °C vs. − 1 °C were not significant (Mann–Whitney U test: Z = −0.456, *p* = 0.65). There were no differences between survival ratios in water vs. wet conditions (Z = −0.116, *p* = 0.91). Among the tested species (Table 1, Fig. 4), survival ratios did not differ between temperatures for *Limnochares aquatica* (H (2, *N* = 14) < 0.001 *p* = 1.0), *Piona nodata* (H (2, *N* = 34) = 3.997, *p* = 0.14), and *Sperchon clupeifer* (H (2, *N* = 9) = 4.145, *p* = 0.13). Survival ratios did differ significantly between temperatures for *Lebertia porosa* (H (2, *N* = 16) = 6.297, *p* = 0.043), *Hygrobates longipalpis* (H (2, *N* = 20) = 9.690, *p* = 0.0079), *Hydrodroma despiciens* (H (2, *N* = 19) = 13.667, *p* = 0.0011), *Lebertia insignis* (H (2, *N* = 13) = 10.508, *p* = 0.0052), *Mideopsis crassipes* (H (2, *N* = 13) = 10.003, *p* = 0.0067), *Piona pusilla* (H (2, *N* = 12) = 8.486, *p* = 0.014) and *Unionicola crassipes* (H (2, *N* = 34) = 21.399, *p* < 0.0001). In all tested species there were no differences in survival ratio at −2 vs. −1 °C (Mann–Whitney U tests all *p* > 0.05).

### Experiment 3

**Fig. 5:**
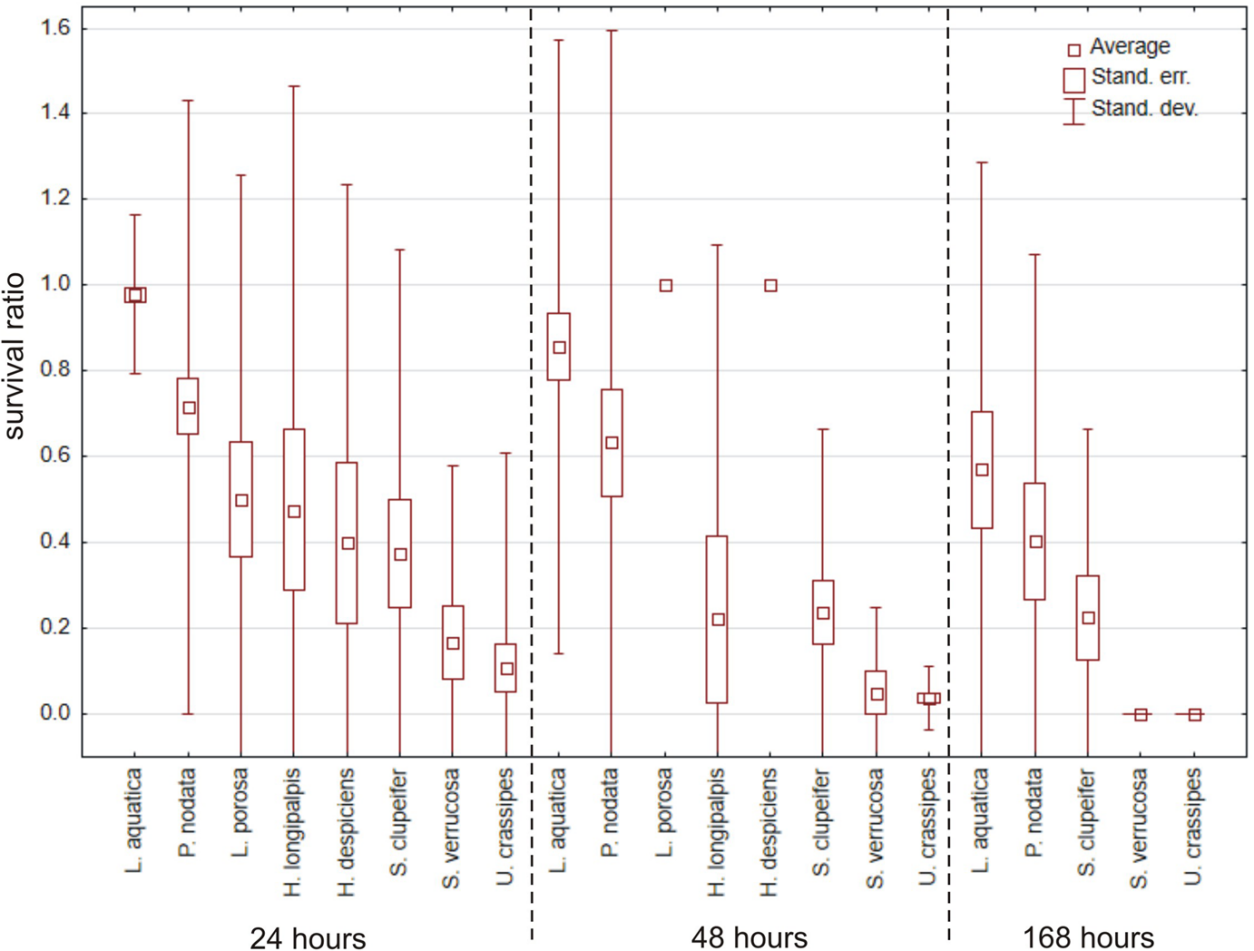
Survival ratio (no. live mites of species X/total no. mites of species X) of freezing by the most numerous species of water mites at −1 °C for 24 h, 48 h and 168 h

**Fig. 6:**
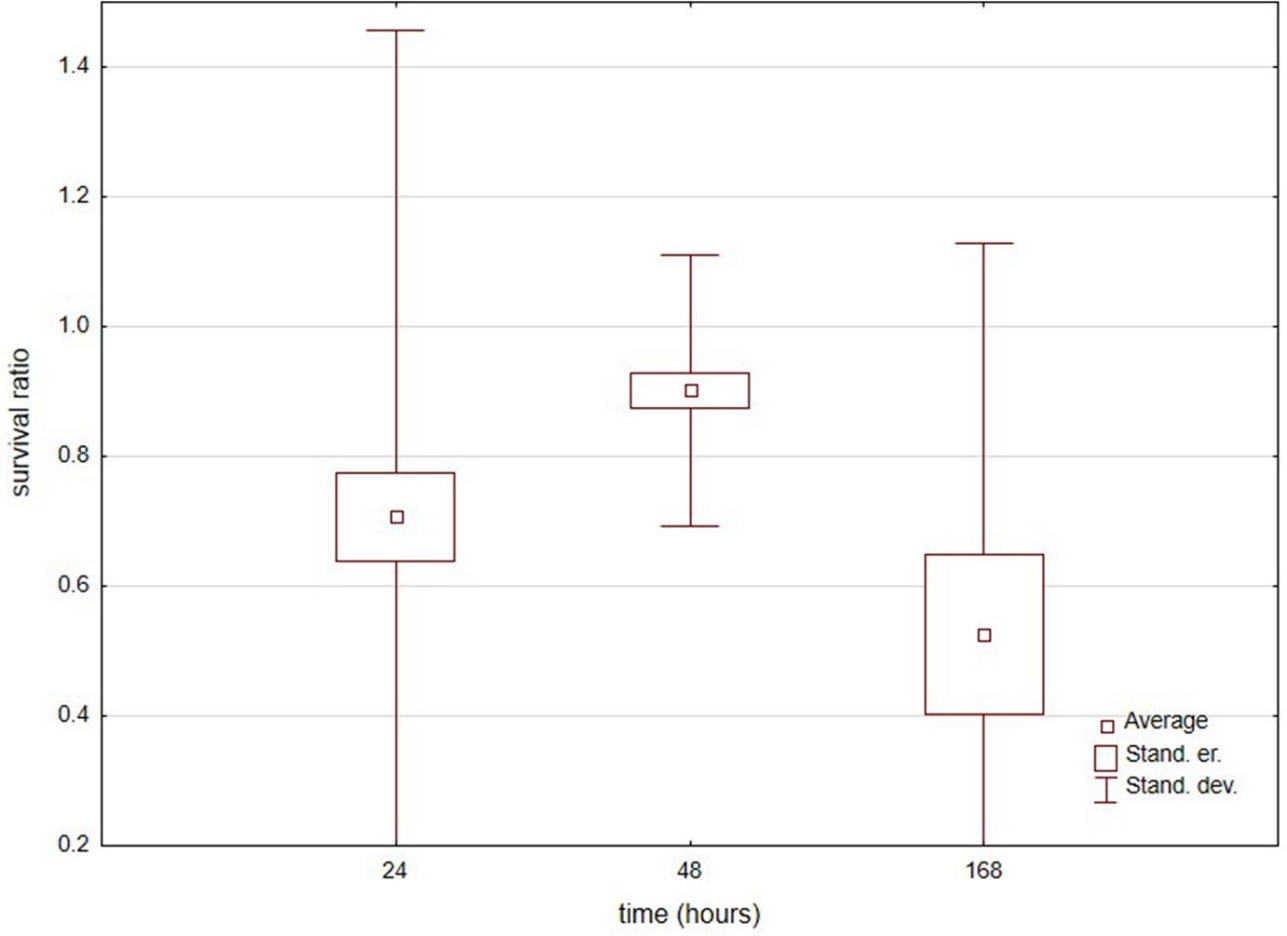
Survival ratio (no. live mites/total no. mites) of water mites at −1 °C for 24 h, 48 h and 168 h

In total, 309 specimens belonging to 15 species (Table 1) were used, eight species had > 10 specimens (Fig. 5). Water mites best survived freezing for 24 h (Fig. 6), next for 48 h, and the worst for 168 h (Kruskal–Wallis test: H (2, *N* = 46) = 9.593, *p* = 0.0083), but survival rates for 24 h and 48 h were very similar (Mann–Whitney U test: Z = 1.693, *p* = 0.27). There were no differences between survival ratios in water vs. wet conditions (Z = 1.296, *p* = 0.19). Among the species only *Limnochares aquatica* was characterized by significant differences (H (2, *N* = 60) = 16.705, *p* = 0.0002).

### Experiment 4

**Fig. 7:**
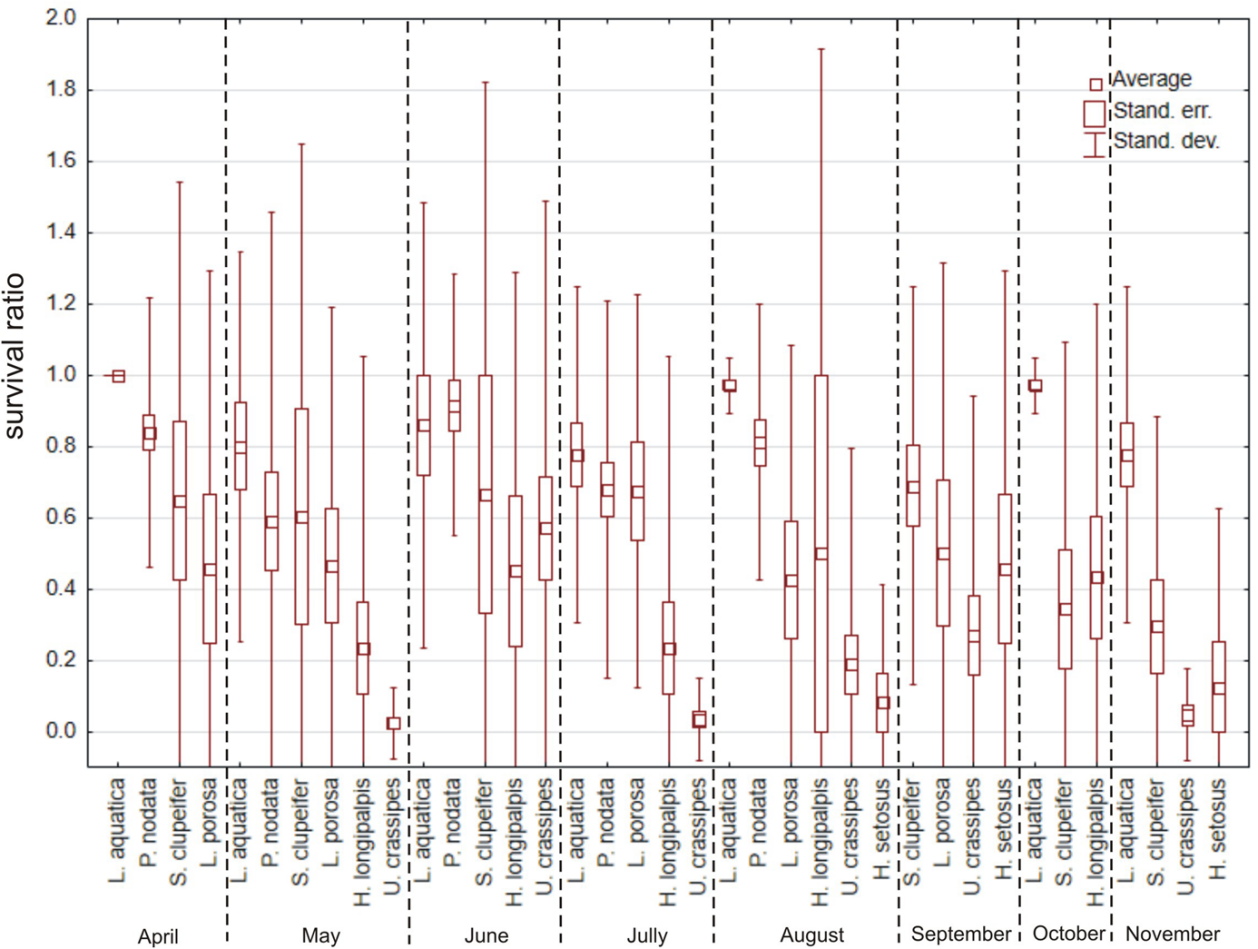
Survival ratio (no. live mites of species X/total no. mites of species X) of freezing (−1 °C) by the most numerous species of water mites collected in different months

**Fig. 8:**
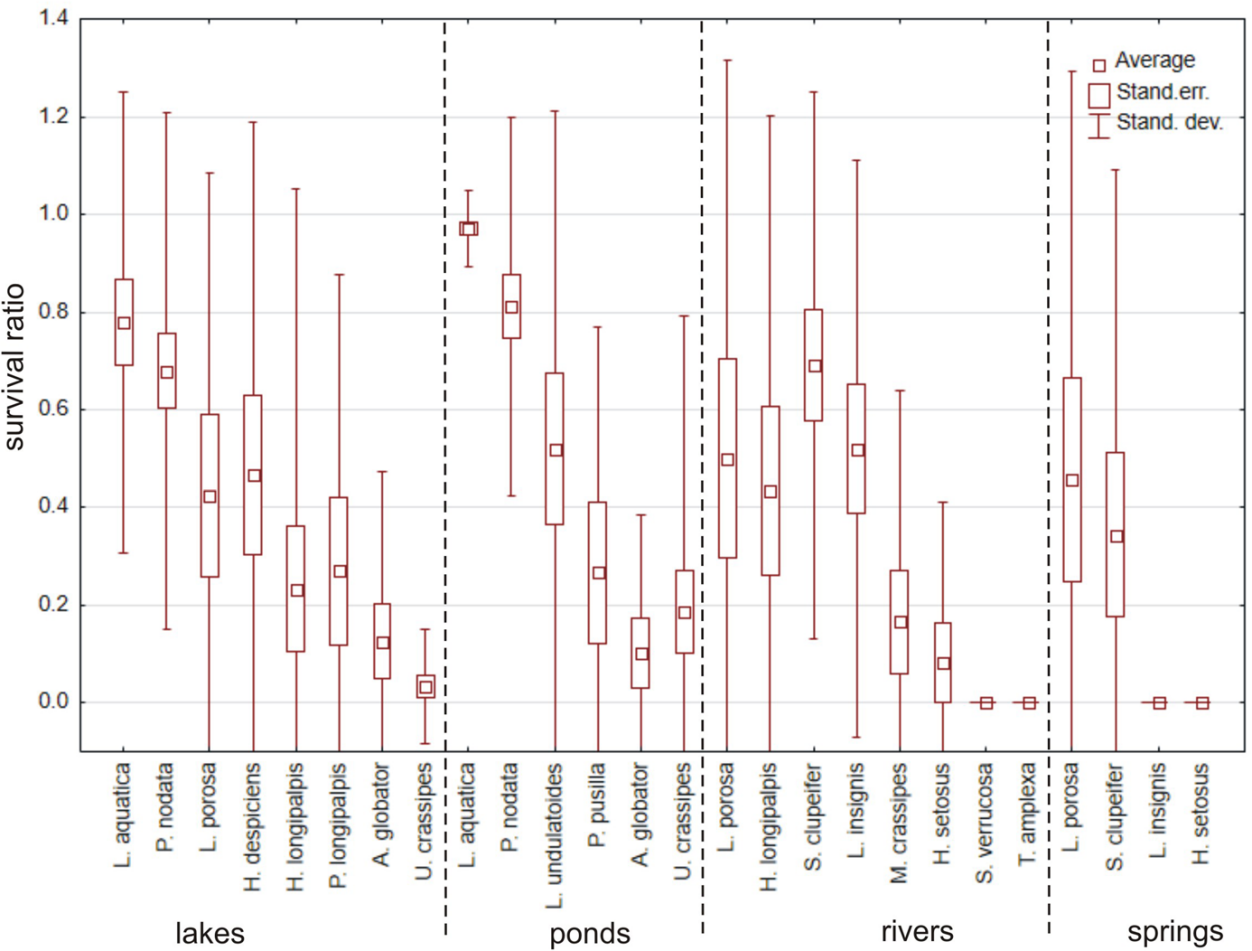
Survival ratio (no. live mites of species X/total no. mites of species X) of freezing (< 0 °C) by particular species of water mites collected in different types of water bodies

**Fig. 9:**
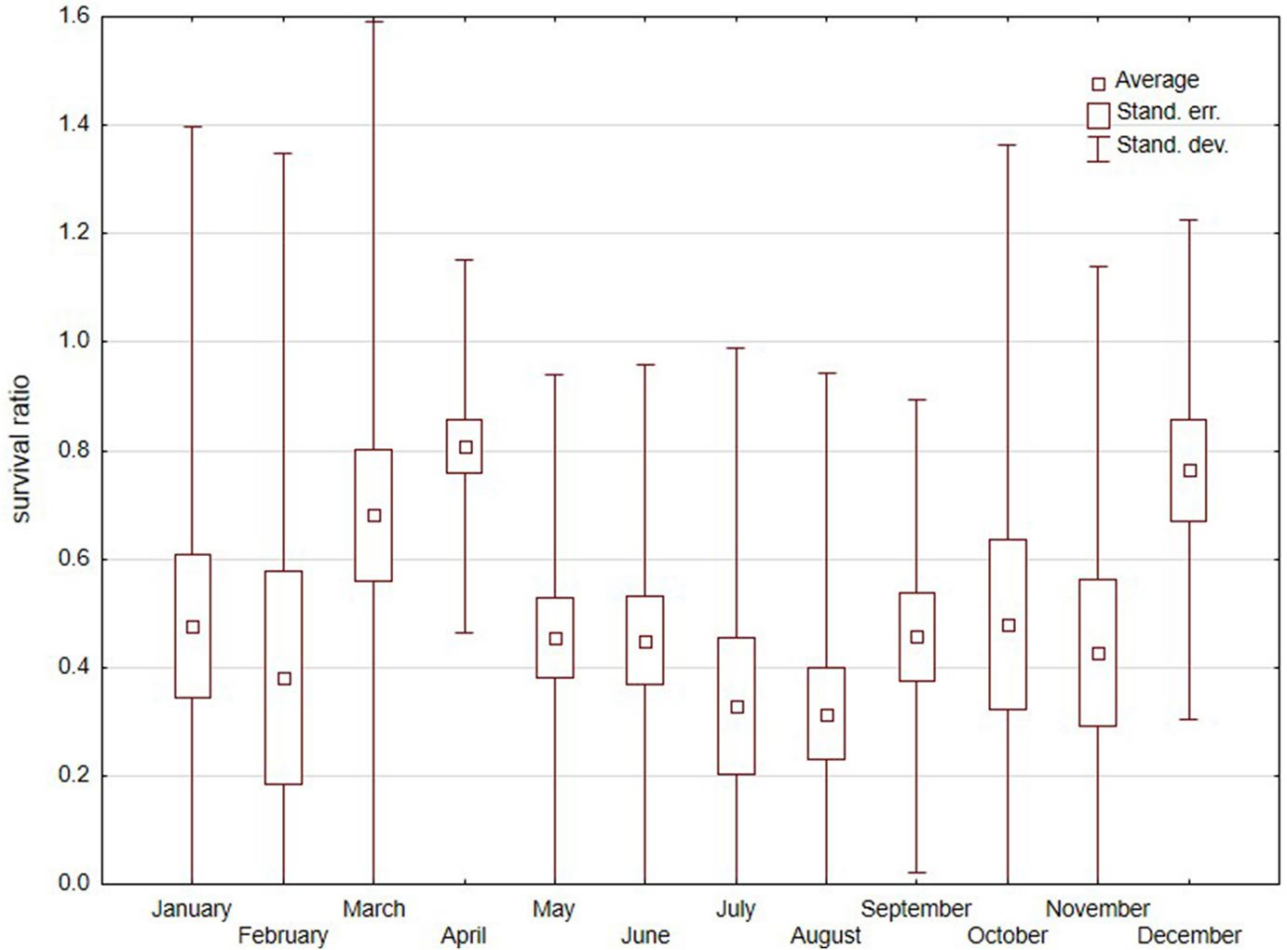
Survival ratio (no. live mites/total no. mites) of freezing (−1 °C) by water mites collected in different months

**Fig. 10:**
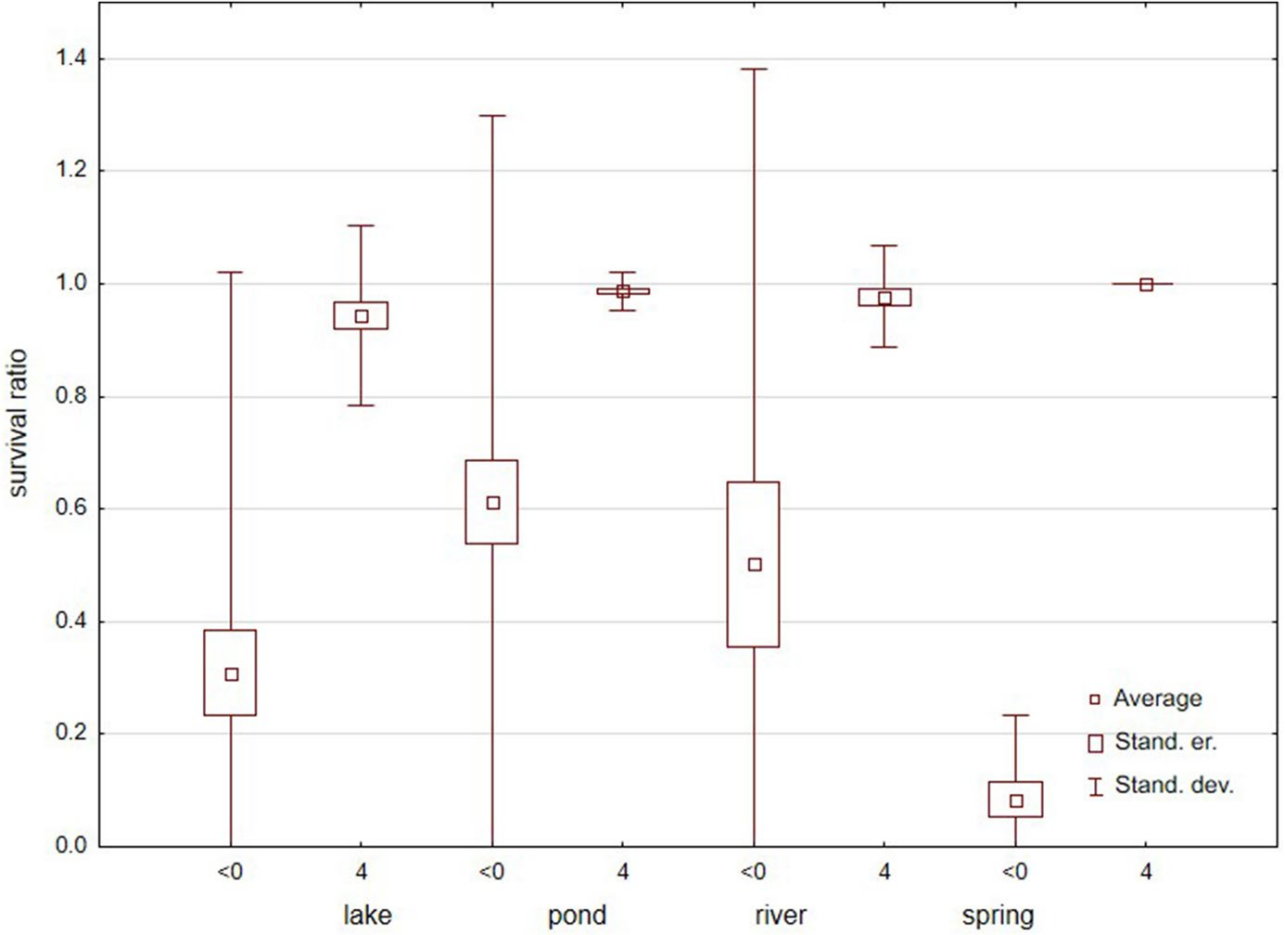
Survival ratio (no. live mites/total no. mites) of freezing by water mites collected in different types of water bodies, at < 0 °C or 4 °C

In total, 1016 specimens belonging to 24 species (Table 1) were used, but only eight species had > 10 specimens (Figs. 7 and 8). There were no significant differences in survival ratios among months of collection (Fig. 9) (Kruskal–Wallis test: H (7, *N* = 65) = 7.708, *p* = 0.36). Among the tested species (Fig. 7) only *Piona nodata* had significant differences (H (4, N = 52) = 12.947, p = 0.012). Mites collected in ponds had the highest survival ratio, followed by mites collected in rivers, lakes and springs (Fig. 10) (H (3, *N* = 58) = 11.237, *p* = 0.011). Among the tested species (Fig. 8) only *Lebertia insignis* had significant differences between streams and springs (Mann–Whitney U test: Z = 2.442, *p* = 0.015).

### Experiment 5

**Fig. 11:**
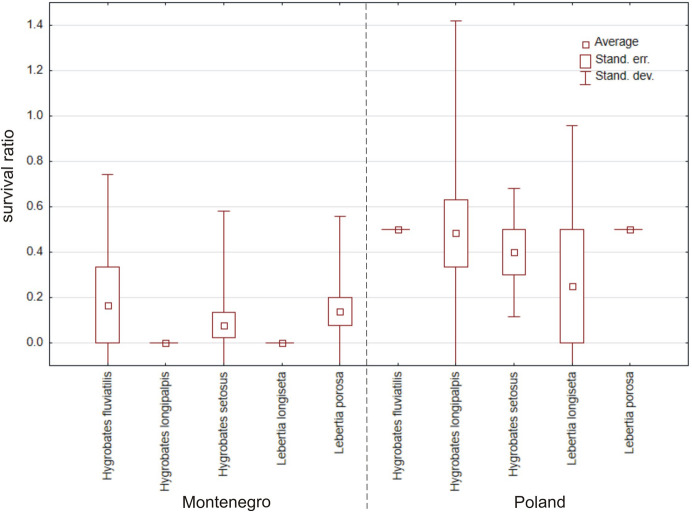
Survival ratio (no. live mites of species X/total no. mites of species X) of freezing (< 0 °C) by particular species of water mites from Montenegro and Poland

**Fig. 12:**
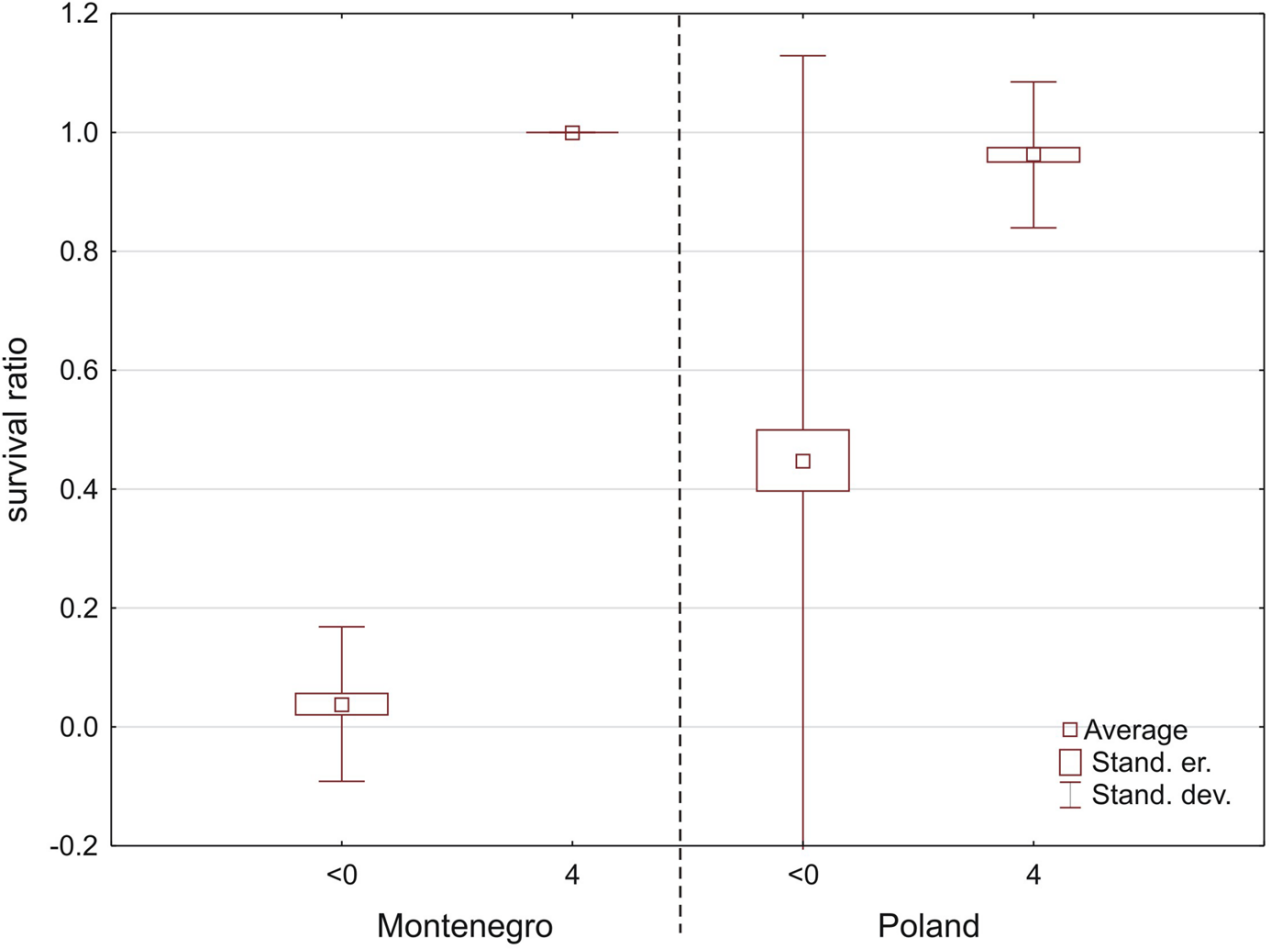
Survival ratio (no. live mites/total no. mites) of freezing by water mites from Montenegro and Poland, at < 0 °C or 4 °C

In total, 225 specimens belonging to five species form Montenegro were tested (Table 1, Fig. 11) and were compared with the same species (110 specimens) from Poland. Overall, mites from Poland had a much higher survival ratio than the ones from Montenegro (Fig. 12) (Mann–Whitney U test: Z = −4.957, *p* < 0.0001). Three of the tested species showed significant differences: *Hygrobates longipalpis* (Z = −1.808, *p* = 0.0071), *H. setosus* (Z = −2.887, *p* = 0.0039) and *Lebertia porosa* (Z = −2.335, *p* = 0.020).

## Discussion

Terrestrial invertebrates are more resistant to sub-zero temperatures than aquatic invertebrates (Ansart and Vernon 2003; Lencioni 2004; Issartel et al. 2006; Novak et al. 2014). This also applies to mites: adult stages of water mites survive temperatures down to −2 °C, in contrast with species of Ixodidae and Tetranychidae, which have been shown to withstand temperatures of about −20 °C (Frisbie and Lee 1997; Khodayari et al. 2013; Yu et al. 2014).

Water mites belong to the cohort Parasitengona, mites with a calyptostase phase, which is mainly found in the imago and deutonymph stages but can also occur in the eggs and larvae (*Eylais extendens*) or only in the larvae (genera *Eylais* and *Hydrachna*) (Wohltmann 2001; Zawal 2002, 2003; Belozerov 2008; Zawal et al. 2018). Nothing is known about the ability of eggs and larvae to survive low temperatures. Depending on the biology of particular species, the deutonymphs are resistant (*Piona* and *Tiphys* genera), the adults (*Arrenurus* (*Arrenurus* s. str.)), or both (*Arrenurus* (*Truncaturus*)) (Wiggins et al. 1980; Smith 1987; Smith et al. 2001). Our results confirm the previous data about owerwintering *Piona nodata* as a deutonymph and *Arrenurus globator* as a deutonymph and adult, but they are in opposition to what was known for *Piona pusilla*: we found adults to be resistant, whereas Smith (1987) mentioned deutonymphs as resistant. The obtained results show species which are characteristic for temporary waters overwinter as deutonymphs (e.g., *Piona nodata*), species from small permanent water bodies overwinter as adults or both as deutonymphs and adults (e.g., *Arrenurus globator*, *Limnochares aquatica*), and species characteristic for lakes and lotic waters overwinter as adults (Table 1).

Survival ratios in water vs. wet conditions were never found to differ significantly, as expected. This may have implications for more detailed experimental work on the impact of freezing water on body injury and survival of water mites.

Several studies on a wide range of taxa, including the plant-inhabiting mite *Tetranychus urticae* and the tick *Haemaphysalis longicornis* (Khodayari et al. 2013; Yu et al. 2014), have confirmed that acclimatization increases species survival at temperatures below 0 °C. In our study on water mites, acclimatization at 4 °C significantly increased survival at temperatures below 0 °C. Acclimatization simulates the natural freezing resistance process generated by a drop in temperature as winter approaches. The large differences in survival between acclimated vs. non-acclimated specimens and the lack of such differences in water mites collected in different months (they were acclimated each time) indicate the effectiveness of acclimatization as a method of simulating the natural process. The high impact of acclimatization on water mite survival concerned species from stagnant waters (*Limnochares aquatica*, *Piona nodata*, *Sperchon clupeifer*, *Arrenurus globator* and *Unionicola crassipes*), with high variability of temperature throughout the year, but it did not apply to species from running waters (*Hygrobates longipalpis*, *Lebertia porosa* and *Sperchonopsis verrucosa*), where the temperature is more or less constant throughout the year. This indicates that acclimatization is an effective method of simulating natural conditions.

The results of our study revealed that the survival of deutonymphs and adults is limited to a small temperature range (−1, −2 °C) and a relatively short freezing time (24 and 48 h). This is probably due to overwintering of water mites in the bottom of reservoirs where temperature drops are not very great, or in the deeper parts of the water where temperature drops only to 4 °C (Viets 1930; Pieczyński 1964; Kowalik 1973; Wiggins et al. 1980; Smith 1987; Zawal et al. 2014), i.e., outside the range of sub-zero temperatures.

The ability to survive low temperatures is a species-specific feature in water mites, as in the case of some aquatic insects (Frisbie and Lee 1997). In our study of water mites with > 10 specimens, the highest survival rates had *Limnochares aquatica*, *Piona nodata* and *Sperchon clupeifer*, followed by *Lebertia porosa*, *L. insignis*, *Hygrobates longipalpis*, *H. setosus*, *Limnesia undulatoides*, *Piona pusilla*, *Arrenurus globator*, *Hydrodroma despiciens*, *Piona longipalpis*, *Sperchonopsis verrucosa*, *Unionicola crassipes* and *Mideopsis crassipes*; no specimens of *Torrenticola amplexa* survived.

The species-specific survival characteristics partially coincide with the survival of water mites from various types of water bodies. In permanent water habitats at the northern temperate latitudes, most species overwinter primarily as inseminated females, in temporary waters as deutonymphs and females, but some taxa can overwinter as eggs and as larvae attached to their host (Smith 1980; Wiggins et al. 1980; Smith et al. 2001). In running waters, lenitophiles and most rheobionts overwinter as eggs or nymphal resting stages, but in many species overwintering of adults can be observed as well (Gerecke 2002; Martin 2010). In some cases, the same species can overwinter in different stages depending on the habitat (Gerecke 2002). Nevertheless, the variety of species occurring in different types of water bodies, and thus their varying ability to survive low temperatures, make it next to impossible to define a relationship between resistance to freezing and the type of water body the water mites are from, despite the fact that water mites from lakes – where they can escape from freezing into deeper parts of water – or from springs – where temperature fluctuations are smaller (lakes and springs) – had the lowest survival rates in temperatures below 0 °C.

Our study showed marked differences in resistance to freezing in water mites from different climatic zones. Water mites from a temperate climate had good survival rates, in contrast to those from a Mediterranean climate, whose survival rates were very poor.

## Conclusions

1. Acclimatization is a high-efficiency method of simulating the natural process by which water mites prepare for overwintering.
2. Water mites survive freezing temperatures to −2 °C, whereas lower temperatures appear to be lethal.
3. Water mite survival is much higher after short (24–48 h) than after long (168 h) freezing times.
4. Freeze resistance seems to be an evolutionary trait of individual species, partly related to the living environment and associated with better survival of various developmental stages.
5. Freezing survival rates are linked to regions of Europe and are much lower in Southern Europe than in Central Europe.
